# Polyacrylonitrile-Polyvinyl Alcohol-Based Composite Gel-Polymer Electrolyte for All-Solid-State Lithium-Ion Batteries

**DOI:** 10.3390/polym14235327

**Published:** 2022-12-06

**Authors:** Yer-Targyn Tleukenov, Gulnur Kalimuldina, Anar Arinova, Nurbolat Issatayev, Zhumabay Bakenov, Arailym Nurpeissova

**Affiliations:** 1 Laboratory of Advanced Materials and Systems for Energy Storage, National Laboratory Astana, Nazarbayev University, 53 Kabanbay Batyr Avenue, Nur-Sultan 010000, Kazakhstan; 2 Department of Mechanical and Aerospace Engineering, School of Engineering and Digital Sciences, Nazarbayev University, 53 Kabanbay Batyr Avenue, Nur-Sultan 010000, Kazakhstan; 3 Department of Chemical and Materials Engineering, School of Engineering and Digital Sciences, Nazarbayev University, 53 Kabanbay Batyr Avenue, Nur-Sultan 010000, Kazakhstan

**Keywords:** lithium-ion battery, polymer electrolyte, NiO anode, conformal coating, Al_2_O_3_ nano additives

## Abstract

The three-dimensional (3D) structure of batteries nowadays obtains a lot of attention because it provides the electrodes a vast surface area to accommodate and employ more active material, resulting in a notable increase in areal capacity. However, the integration of polymer electrolytes to complicated three-dimensional structures without defects is appealing. This paper presents the creation of a flawless conformal coating for a distinctive 3D-structured NiO/Ni anode using a simple thermal oxidation technique and a polymer electrolyte consisting of three layers of PAN-(PAN-PVA)-PVA with the addition of Al_2_O_3_ nanoparticles as nanofillers. Such a composition with a unique combination of polymers demonstrated superior electrode performance. PAN in the polymer matrix provides mechanical stability and corrosion resistance, while PVA contributes to excellent ionic conductivity. As a result, NiO/Ni@PAN-(PAN-PVA)-PVA with 0.5 wt% Al_2_O_3_ NPs configuration demonstrated enhanced cycling stability and superior electrochemical performance, reaching 546 mAh g^−1^ at a 0.1 C rate.

## 1. Introduction

With the development of energy storage applications, such as portable electronic devices, electric vehicles, and the growing need for storage of new green energy (solar, tide energy, wind, nuclear, biomass, etc.) in smart grids, the demand for lithium-ion batteries (LIBs) has recently been increasing rapidly [1,2,3]. To meet the performance requirements of some new advanced applications, many research works have shifted their attention to enhancing power density [4,5,6]. In order to obtain a high energy density for batteries, the electrode surface area may be increased to permit large mass loading of electrochemically active material per unit area and the development of new electrode materials. Traditional planar or two-dimensional (2D) electrode design places a limit on the active material mass loading inside a 2D surface, but it can be expanded by using thicker electrodes. The electrode thickness is nevertheless constrained by the following factors: risks of a thick electrode detaching from the current collector during repeated charge-discharge cycles; slow diffusion/charge and mass transfer of lithium-ion (Li^+^) through a thick electrode layer; and, in the case of lithium (Li) metal-based batteries, the electrode’s development of Li dendrites, which can short-circuit the battery [7,8]. The three-dimensional (3D) batteries concept is a result of the solution of these issues and the need to increase the areal capacity of batteries. A highly developed 3D surface allows for a larger amount of active material to be deposited while maintaining a smaller thickness, thus avoiding the challenges associated with a thick electrode [4]. Along with these advantages, a 3D configuration can compensate for and mitigate an electrode’s volume variations due to Li^+^ insertion and extraction [9].

The mechanical characteristics of a separator/electrolyte and its conformal deposition are critical for ensuring the electrical separation of the electrodes in 3D batteries with a solid polymer electrolyte. Gel polymer electrolytes (GPEs) are the ideal electrolytes for complex three-dimensional configurations because they may function as both electrolytes and separators, reducing the leakage of liquid electrolytes and the high interface resistance of solid electrolytes. In order to reduce the Li^+^ diffusion distance and enhance charge transfer, the electrolyte should be flexible enough to tolerate electrode expansion while still being as thin as is practical [10,11,12]. A number of methods for conformal deposition of polymer-based electrolytes onto 3D electrodes were studied and reported. Numerous researchers have made an effort to create GPE for 3D batteries using electropolymerization [13,14,15,16].

GPEs are limited by temperature because of their high crystallinity at room temperature, which results in low conductivity [17]. The solid–solid interface of batteries becomes greater than the solid–liquid interface as a result of insufficient electrode–electrolyte contact, which also causes increased polarization and slower ion transport. Additionally, during charge and discharge cycles, the volume change of the electrodes sped up the breakdown of the contact between the electrodes and electrolytes. GPEs have a number of benefits, such as excellent Li dendrite inhibition, wide electrochemical window and high safety [18]. In terms of shape flexibility and manufacturing processability, GPEs have more advantages [19]. Due to the poor segmental mobility of their polymer chains, GPEs still need a long way for their industrial application because of their low Li^+^ conductivity.

Some problems such as investigating new electrolyte compositions to control the growth of lithium metal or changing the solid electrolyte interphase (SEI), which resulted in the improvement of Li^+^ conductivity, have been addressed in the past in a variety of ways [20,21,22]. Lithium nitrate (LiNO_3_) [23,24], halogenated lithium salt (LiX) [25,26] or gas-phase dopants [27] are a few examples of the additives that have been added to the electrolyte to modify the SEI layers in order to build a robust SEI on lithium metal. As an alternative, polymers [28,29,30,31], ceramic nanoparticles [32,33,34] and carbon-based materials [22,35] were used to precoat an artificial SEI layer on the surface of lithium metal anodes. Particularly, different polymer-based layers that could act as a protective SEI layer for lithium metal anodes were studied because of their mechanical properties, chemical inertness, and scalable/cheap manufacturing process. Examples include nanoporous poly(dimethylsiloxane) films [36] and a polymer network with a single conducting ion [37]. To increase the ion conductivity and obtain electrochemically stable and high-rate performance all-solid-state LIBs, extensive research has been carried out on polymers, such as polyacrylonitrile (PAN) and polyvinyl alcohol (PVA) and only a few works are devoted to the combined use of PAN and PVA [38,39,40].

In this paper, porous NiO was grown by thermal oxidation on the surface of Ni foam and used as an anode without further treatments. Ni foam’s unique structure provides good electric conductivity, high mesoporosity, tremendous surface area and excellent chemical stability in a range of liquid electrolytes [41]. Layer-by-layer dip-coating technique was employed to obtain three polymer layers of PAN-(PAN-PVA)-PVA to fabricate a polymer electrolyte perfect enough to conformally coat the Ni foam structure without defects. PAN was utilized as the first polymer layer host matrix and a gelating agent. Li^+^ mobility is aided by the gelating agent, which holds the liquid electrolytes [42]. From a stress and strain perspective, PAN-based electrolytes are thought to be optimal for creating a good interface with electrodes [43]. It has been found that PAN-based electrolytes can enhance battery performance and improve the adhesive/cohesive strength within the electrode composite [44]. Additionally, the PAN-hosted polymer exhibits exceptional qualities such as good compatibility with the Li electrodes, high ionic conductivity, high thermal stability, good morphology for electrolyte uptake and the ability to reduce the dendrite formation during the charging/discharging process in LIBs [45]. PVA layer was used to have stable interfacial contact with an electrode material. It demonstrates the excellent mechanical flexibility of the gel as well as the remarkable reversibility of Li^+^ [46].

This paper reports a perfect conformal coating of a unique 3D structured NiO/Ni anode synthesized by a simple thermal oxidation process with three polymer electrolyte consisting of three layers of PAN-(PAN-PVA)-PVA with the addition of Al_2_O_3_ nanoparticles (NPs) as nanofillers. A 3D anode may be coated uniformly and steadily with a polymer using the sol-gel dip-coating process. This coating could then be dried and loaded with a liquid electrolyte to create Li^+^ conductive GPE. With a capacity retention of 96% and coulombic efficiency of 97%, the 3D NiO/Ni@PAN-(PAN-PVA)-PVA with 0.5 wt% Al_2_O_3_ NPs configuration demonstrated stable cycleability up to 100 cycles.

## 2. Experimental Part

### 2.1. Material Preparation

Anode material was synthesized from commercially available nickel (Ni) foam (Sigma Aldrich Chemie GmbH, Steinheim, Germany) (thickness 0.9 mm, bulk density 0.62 g cm^−3^, porosity 93%). Ni foam with a thickness of 0.9 mm was cut into round shapes with 14 mm diameter. Then, they were rinsed with absolute ethanol and acetone (Sigma Aldrich Chemie GmbH, Riedstrasse 2, Steinheim, Germany) (1:1) in an ultrasonic bath (Elma Schmidbauer GmbH, Singen, Germany) for 1 h to remove the surface impurities, then dried in a furnace (Memmert GmbH, Schwabach, Germany) at 60 °C. After drying, NiO/Ni anode was fabricated by thermal oxidation at 700 °C for 5 min [47]. Then, three polymer layers PAN-(PAN-PVA)-PVA were coated on NiO/Ni anodes by a sol-gel dip-coating method. In a typical procedure, PAN and PVA were dissolved in N,N-Dimethylformamide (DMF) (Sigma Aldrich Chemie GmbH, Steinheim, Germany) and deionized water (DI water), respectively. The polymer solutions were then stirred with a magnetic stirrer at 600 r min^−1^ for 6 h. First, the cleaned NiO/Ni anode was dipped in the PAN (2 wt%) dissolved in DMF solution for 10 s and then removed and dried in a vacuum oven at 60 °C for 2 h. The second layer was PAN-PVA. The dried NiO/Ni anode was coated with a layer of PAN-PVA by dipping in PAN-PVA (1 wt%) dissolved in DMF solution for 10 s and then removed and dried in a vacuum oven at 60 °C for 2 h. The third layer of PVA was obtained by dipping in PVA (2 wt%) dissolved in DMF:DI water (7:3) solution for 10 s and then removed and dried in a vacuum oven at 60 °C for 2 h. Moreover, the different concentrations of Al_2_O_3_ (0.25, 0.5 wt%) (Sigma Aldrich Chemie GmbH, Steinheim, Germany) were mixed with PAN, PAN-PVA, and PVA polymer solutions using a magnetic stirrer for 12 h to form a homogeneous solution. The experiment was repeated from the first step.

### 2.2. Materials’ Characterization

The crystal structures of the obtained NiO/Ni anodes were analyzed using XRD (SmartLab, Rigaku Co., Takatsuki, Japan, Cu Kα radiation, λ = 0.154056 nm). The XRD data were obtained over a 2θ range from 20 to 80 °C at a scan rate of 6 deg. min^−1^ using 40 kV, 30 mA X-ray. Scanning electron microscopy coupled with energy-dispersive X-ray spectroscopy (SEM-EDS, JSM-7500F, JEOL Ltd., Yamagata, Japan) were employed to investigate the morphology and homogeneity of the distribution of NiO/Ni anode components. Structural modifications in GPE were analyzed using Fourier-transform infrared spectroscopy (FTIR, Nicolet iS10 FT-IR Spectrometer, Thermo Fisher Scientific Inc., Ogden, UT, USA).

### 2.3. Electrochemical Investigation

The electrochemical performance of NiO/Ni@PAN-(PAN-PVA)-PVA anodes was investigated using the CR2032-type coin cells assembled in an argon-filled glovebox (M. Braun Inertgas-Systeme GmbH, Gerlingen, Germany). Metal Li was used as both counter and reference electrodes. A Celgard 2400 microporous polypropylene membrane was used as a separator. The electrolyte was 1 M LiPF_6_ in a mixture of ethylene carbonate/ethyl-methyl carbonate/dimethyl carbonate (EC/EMC/DC, 1:1:1 vol%). The polymer film on the surface of the anode was activated by adding 4–5 drops of 1 M LiPF_6_ electrolyte solution of EC, EMC and DC (1:1:1 (*v*/*v*) ratio). The coin cells were tested galvanostatically on a multi-channel battery testing system (BT-2000, Arbin Inc., Hong Kong, China and Neware Battery tester, Neware Co., Hong Kong, China) at a current density of 0.1 C, between the cut-off potentials of 0.01 and 3.0 V. Cyclic voltammetry (CV) was performed using a VMP3 potentiostat/galvanostat (Bio-Logic Science Instrument Co., Seyssinet-Pariset, France) at the scan rate of 0.1 mV s^−1^ between 0.01 and 3.0 V.

## 3. Results and Discussions

**Figure 1:**
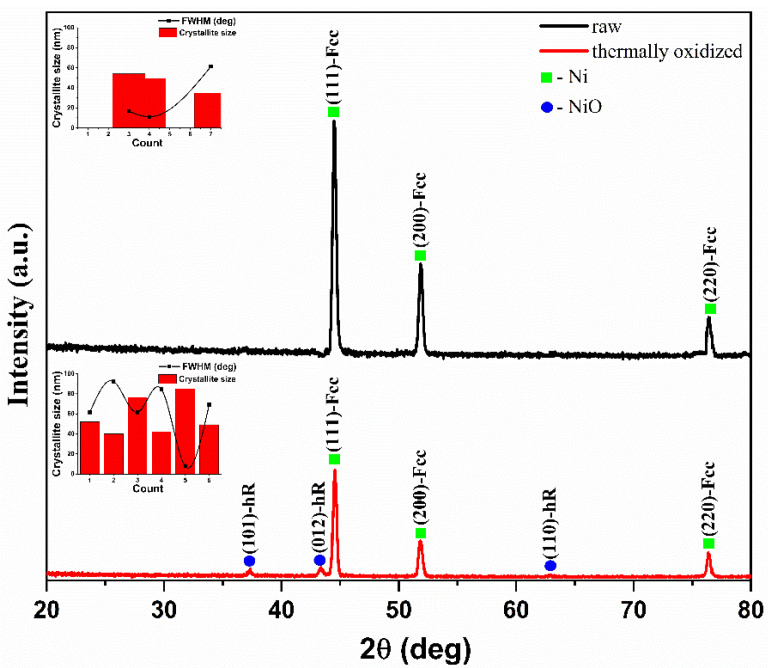
XRD patterns of NiO/Ni thermally oxidized at 700 °C for 5 min compared with the standard peak for Ni-foam.

To verify the purity of the phase, the as-prepared NiO/Ni anode was analyzed by XRD analysis. The XRD patterns for pristine Ni foam and thermally oxidized Ni foam at 700 °C for 5 min. are shown in Figure 1. The peaks of thermally oxidized Ni foam correspond to NiO, indicating the successful formation of NiO on the surface of Ni foam. The face-centered cubic Ni crystal planes are demonstrated by the three sharp peaks of the pure Ni foam, which are located at 2θ = 44.5°, 51.8°, and 76.4° (JCPDS No. 01-071-4654). In contrast, the diffraction peaks of the NiO/Ni-foam are observed at 37.4°, 43.4°, and 62.9°, which can be assigned to the (101), (012), and (110) planes of rhombohedral NiO with space group R-3m, respectively (JCPDS card No. 00-044-1159). Impurity phases were not detected, indicating the successful preparation of NiO [48,49]. The average sizes of Ni and NiO crystallites were calculated using the Paul Scherrer equation, 50.97 nm and 59.10 nm, respectively.

**Figure 2:**
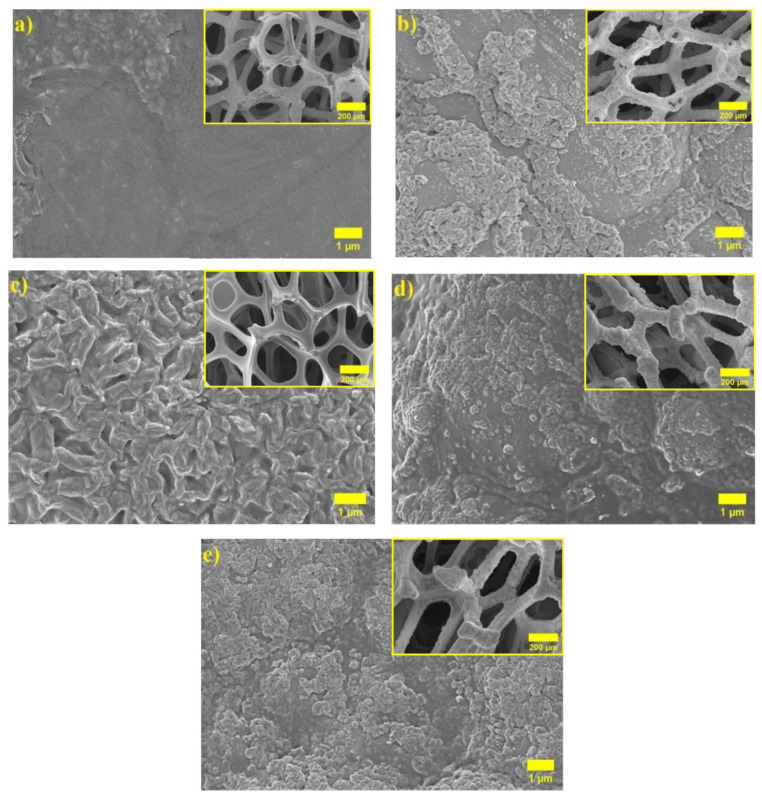
SEM images for (**a**) pristine Ni foam, (**b**) after thermal oxidation, (**c**) NiO/Ni@PAN-(PAN-PVA)-PVA, (**d**) NiO/Ni@PAN-(PAN-PVA)-PVA with the addition of 0.25% Al_2_O_3_, and (**e**) NiO/Ni@PAN-(PAN-PVA)-PVA with the addition of 0.5 wt% Al_2_O_3_.

Figure 2a,b present the SEM images of the pristine Ni foam and NiO/Ni foam. A 3D porous structure with uniformly spaced microscale pores is present in the pristine Ni foam (Figure 2a). It can be observed that after thermal oxidation, the surface of the struts of Ni foam became rough (Figure 2b) due to NiO growth. Further, after coating with PAN-(PAN-PVA)-PVA polymer layers, the aggregates of the cauliflower shape were observed, which can be observed in the inset of Figure 2c–e, showing morphology after the addition of Al_2_O_3_ nanofillers and the surface roughness increases by the insolubility properties of Al_2_O_3_ and the formation of grains on the surface. Since the PAN-(PAN-PVA)-PVA polyelectrolyte coating loaded with 1 M LiPF_6_ solution in EC/EMC/DC is intended to act as an electrolyte providing Li^+^ conductance while ensuring electrical insulation of electrodes from each other, the absence of any cracks or holes in the polymer coating is essential for the safe operation of the battery [50].

**Figure 3:**
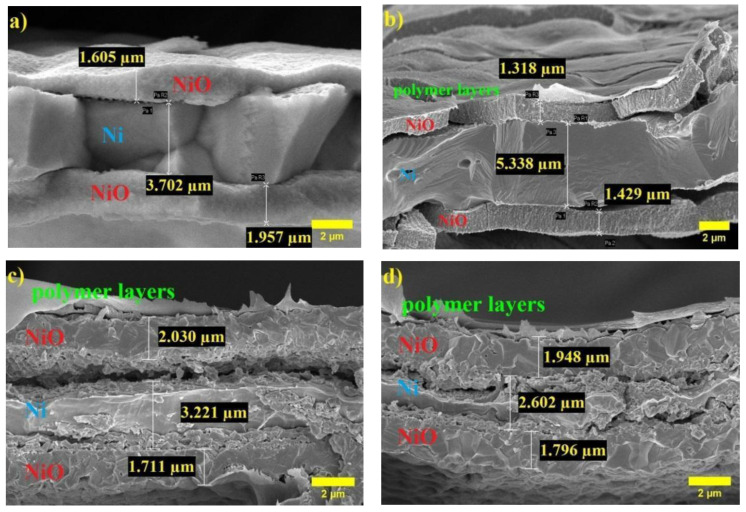
Cross-sectional SEM images for (**a**) NiO/Ni foam, (**b**) NiO/Ni@PAN-(PAN-PVA)-PVA, (**c**) NiO/Ni@PAN-(PAN-PVA)-PVA with the addition of 0.25 % Al_2_O_3_, and (**d**) NiO/Ni@PAN-(PAN-PVA)-PVA with the addition of 0.5 wt% Al_2_O_3_.

A cross-sectional SEM analysis was also carried out to measure the thickness of the NiO and coated polymer layers. Figure 3b reveals that the surface of Ni foam was successfully covered with the NiO layer inside and outside with thicknesses in the range of ~1.605 and 1.957 μm. As shown in Figure 3b, the total thickness of the 3 layers is 0.0492 μm. The addition of Al_2_O_3_ in the amount of 0.25 and 0.5 wt% in the coating further increased the layers’ thickness by almost 1.5 and 5.1 times, 0.07498 μm and 0.253 μm, respectively (Figure 3c,d).

**Figure 4:**
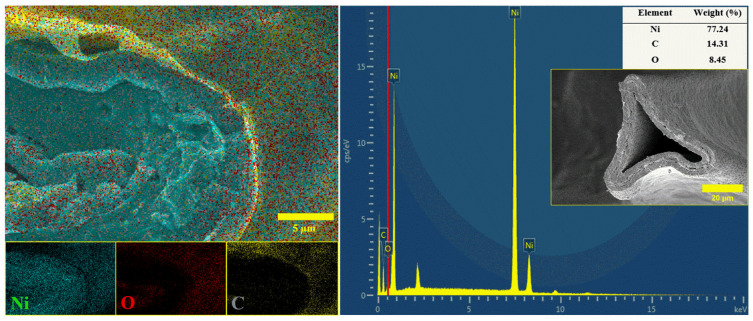

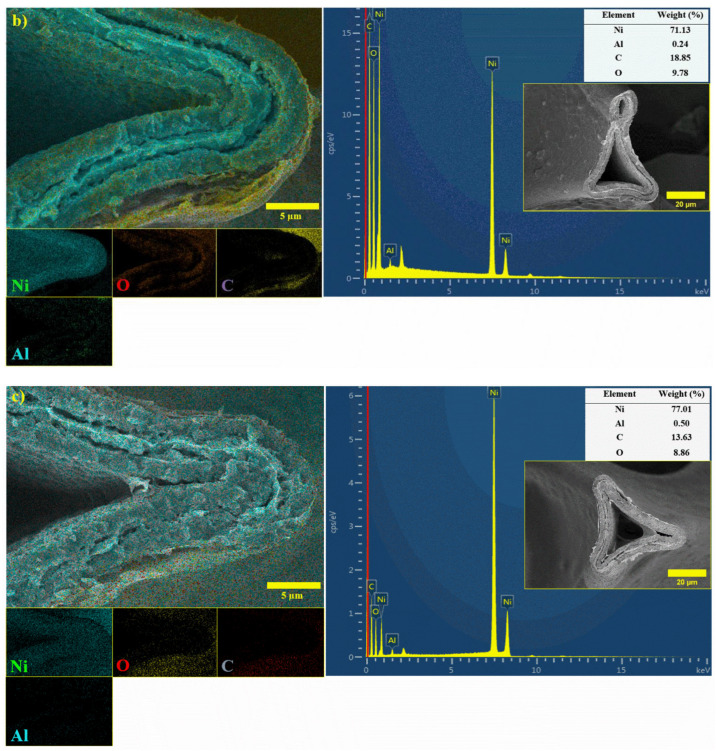
Cross-sectional SEM images and EDS analysis for (**a**) NiO/Ni@PAN-(PAN-PVA)-PVA, (**b**) NiO/Ni@PAN-(PAN-PVA)-PVA with the addition of 0.25 wt% Al_2_O_3_, and (**c**) NiO/Ni@PAN-(PAN-PVA)-PVA with the addition of 0.5 wt% Al_2_O_3_.

To confirm the coating of polymers and uniform distribution of Al_2_O_3_ NPs on the surface of NiO/Ni foam, the EDS analysis was acquired. The results are shown in Figure 4, where polymer coatings with Al_2_O_3_ reveal a uniform distribution of elements, as expected.

**Figure 5:**
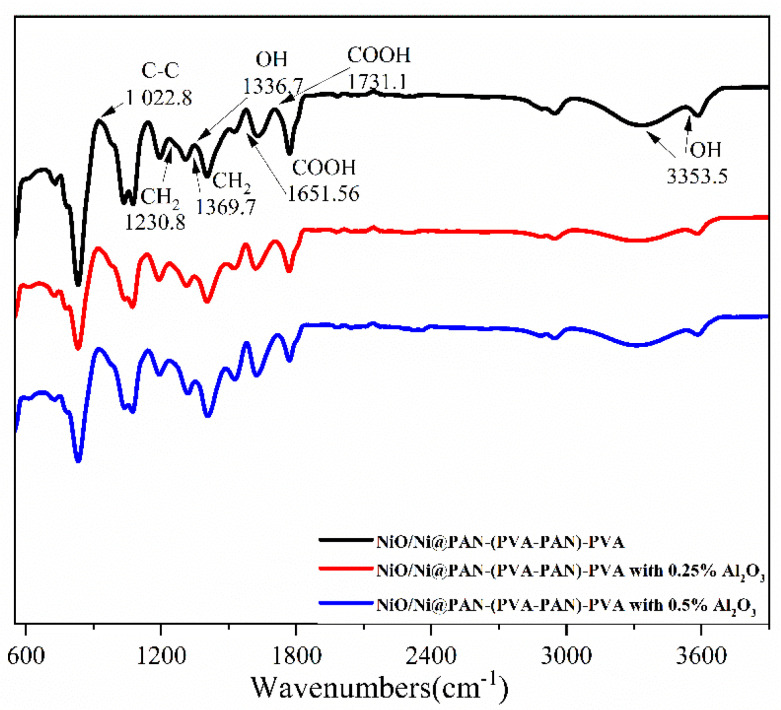
FTIR spectra for NiO/Ni coated with PAN-(PVA-PAN)-PVA layers and Al_2_O_3_ nanofillers.

FTIR results confirm the presence of polymers PAN and PVA layers after dip-coating. The resulting spectra have typical peaks between 400–3600 cm^−1^ attributed to PAN and PVA, as shown in Figure 5. The presence of peaks at 2326 cm^−1^ and 1447 cm^−1^ is characteristic of groups -C≡N, -O-CH_3_, while peaks at 1057–1394 cm^−1^ are a series of bands corresponding to the vibrations of esters for the chemical structure of PAN. The distinct groups for the PVA broadband are observed in ~2900–3500 cm^−1^ due to stretching-bound vibrations of -OH groups. Absorption in ~1600 and ~1454 cm^−1^ is due to scissor, pendulum, and skeletal stretching vibrations of hydrocarbons with an unbranched chain of CH_2_ group bonds.

**Figure 6:**
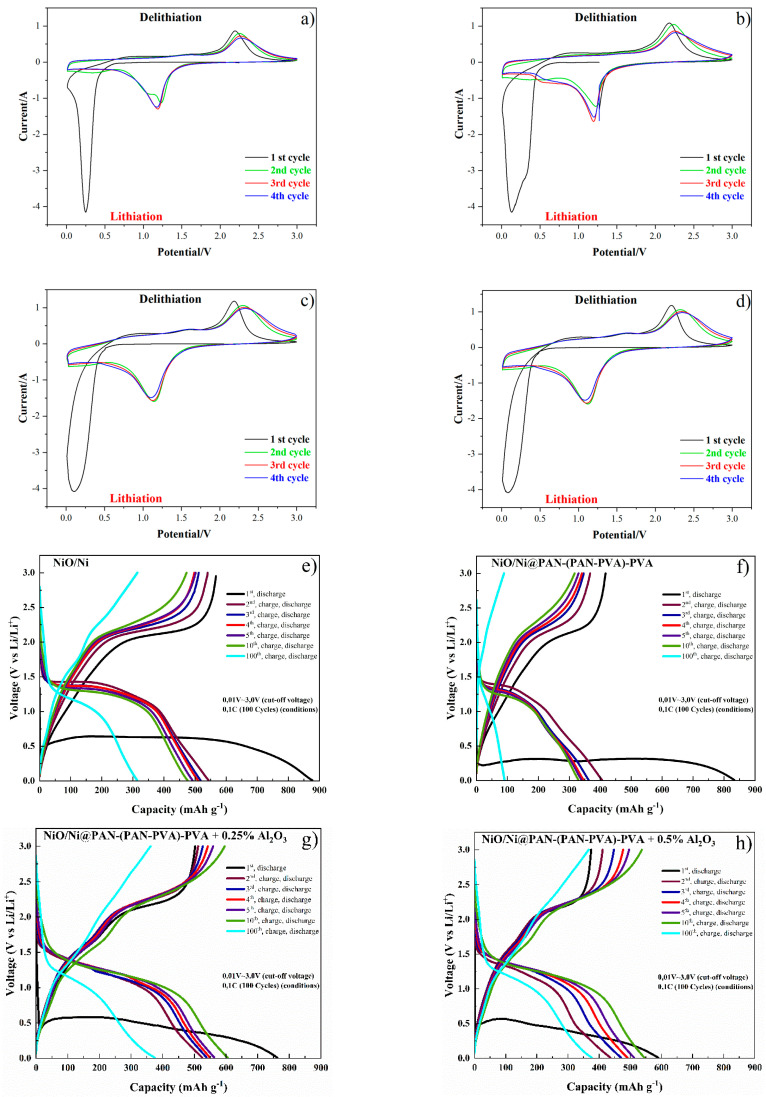

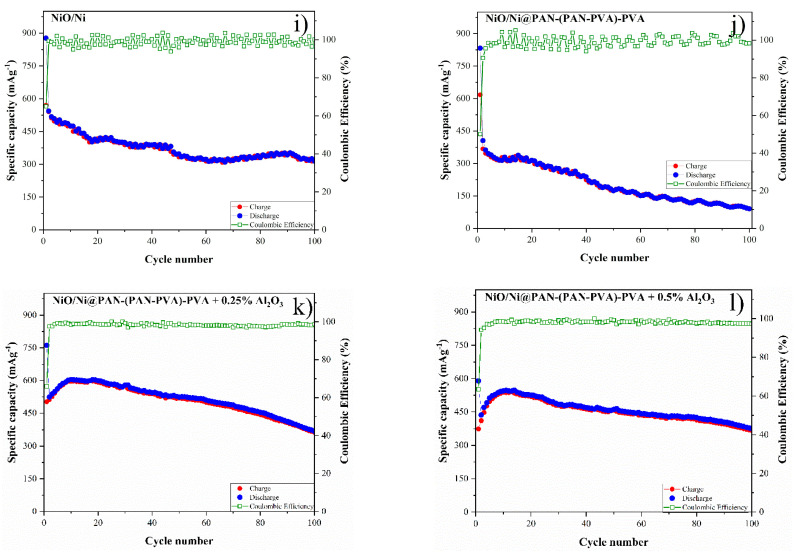
CV plateaus and charge–discharge curves of (**a**,**e**) NiO/Ni foam, (**b**,**f**) NiO/Ni@PAN-(PAN-PVA)-PVA, (**c**,**g**) NiO/Ni@PAN-(PAN-PVA)-PVA with addition of 0.25 wt% Al_2_O_3_, (**d**,**h**) NiO/Ni@PAN-(PAN-PVA)-PVA with addition of 0.5 wt% Al_2_O_3_ electrodes. Cycle performance for 100 cycles of (**i**) NiO/Ni foam, (**j**) NiO/Ni@PAN-(PAN-PVA)-PVA, (**c**) NiO/Ni@PAN-(PAN-PVA)-PVA with addition of 0.25 wt% Al_2_O_3_ (**k**), NiO/Ni@PAN-(PAN-PVA)-PVA with the addition of 0.5 wt% Al_2_O_3_ electrodes (**l**).

Furthermore, CV measurements were conducted at a scanning rate of 0.1 mV s^−1^ within a potential window between 0.01 and 3.0 V to investigate the electrochemical activity of the produced electrodes. CV profiles of NiO/Ni foam and NiO/Ni@PAN-(PAN-PVA)-PVA electrodes with and without Al_2_O_3_ are shown in Figure 6a–d. CV for NiO/Ni foam electrodes shows that there is just one redox couple peak related to the oxidation of Ni^2+^ [51]. The electrodes exhibit anodic and cathodic peaks at 2.18–2.29 V and 0.09–0.25 V, respectively. The formation of amorphous Li_2_O, the formation of a partially reversible solid electrolyte interphase (SEI) film, and the reduction of NiO to Ni are responsible for the first cycle’s prominent cathodic peak at about 0.09–0.25 V [52]. The cathodic peak became broader in the subsequent cycles and shifted to about 1.2 V for all electrodes. The performance of NiO electrodes as reported in the literature [53] is in agreement with the anodic peaks, which have essentially not changed. It is important to note that the addition of 0.25 and 0.5% Al_2_O_3_ did not significantly change the peak potentials of any electrodes, but did cause the peak intensities of NiO/Ni@PAN-(PAN-PVA)-PVA and NiO/Ni@PAN-(PAN-PVA)-PVA to slightly rise. The peak intensities for NiO/Ni@PAN-(PAN-PVA)-PVA and NiO/Ni@PAN-(PAN-PVA)-PVA with the addition of 0.25 and 0.5% Al_2_O_3_ increased very marginally; however, it is important to note that the peak potentials of all electrodes remained similar. The most stable sample is defined as NiO/Ni@PAN-(PAN-PVA)-PVA with the addition of 0.5 wt% Al_2_O_3_. The overall lithiation and delithiation can be represented in Equations (1) and (2).

The NiO anode’s electrochemical reactions during charge–discharge processes [54,55]:

NiO + 2Li^+^ + 2e^−^ → Ni + Li_2_O, (1)

Ni + Li_2_O → NiO + 2Li^+^ + 2e^−^ (2)

Figure 6e–h shows the discharge curves of NiO/Ni foam and NiO/Ni@PAN-(PAN-PVA)-PVA, NiO/Ni@PAN-(PAN-PVA)-PVA with the addition of 0.25, 0.5 wt% Al_2_O_3_ in the initial 10 cycles and at the 100th cycle. An extended voltage plateau can be observed at around 0.25–0.4 V for all samples in the 1st discharge cycle, resulting from NiO reduction to Ni [56]. During the charge cycle, a voltage plateau of 2.0–2.7 V can be observed, which corresponds to the reversible process of NiO production from Ni and Li_2_O. With the obtained CV profiles, the results are in good agreement.

Figure 6i–l represents the cycle performance of prepared electrodes for 100 cycles between 0.01 and 3.0 V. Initially, the cyclic performance of the cell was studied at a current density of 0.1 C. The reversible capacity of NiO/Ni and NiO/Ni@PAN-(PAN-PVA)-PVA electrodes is about 400 mAh g^−1^ and 150 mAh g^−1^ with approximately 99% Coulombic efficiencies. For the NiO/Ni@PAN-(PAN-PVA)-PVA electrode, the charge and discharge capacities maintain 581 mAh g^−1^ and 856 mAh g^−1^ during the 1st cycle, respectively. However, it then decreases dramatically after the 1st cycle due to the poor ionic conductivity of PVA and PAN. For NiO/Ni and NiO/Ni@PAN-(PAN-PVA)-PVA, the capacities decrease gradually after 2 cycles. The capacity of NiO/Ni foam electrodes, on the other hand, increases after 18 cycles and varies up to 100 cycles. The NiO/Ni@PAN-(PAN-PVA)-PVA electrode’s capacity increases up to the 18th cycle, then gradually decreases to a minimum of 75 mAh g^−1^ in the 100th cycle. Relatively uniform reduction in capacity for NiO/Ni@PAN-(PAN-PVA)—the diffusion of Li^+^—explains the PVA electrode through the pores of the polymer. With the addition of Al_2_O_3_, only a gradual decrease in capacity was observed (Figure 6k–l).

The irreversible capacity increase during the first 10 cycles might be related to the influence of insoluble Al particles on the SEI formation (Figure 6k–l). Cycle performance curves for NiO/Ni@PAN-(PAN-PVA)-PVA with the addition of 0.25 and 0.5 wt% Al_2_O_3_ are similar up to the 10th cycle with the subsequent decrease. However, NiO/Ni@PAN-(PAN-PVA)-PVA with the addition of 0.5 wt% Al_2_O_3_ is more stable and reaches the minimum capacity of 395 mAh g^−1^ in the 100th cycle. The cell with the GPE and the addition of 0.5 wt% Al_2_O_3_ has constant coulombic efficiency, which was close to 100% in the overall battery operation. Coating NiO/Ni with a dielectric polymer layers can efficiently act as a capsule, which mitigates the volume expansion of the electrode. Thus, the capacity retention of the NiO/Ni@PAN-(PAN-PVA)-PVA with 0.5 wt% Al_2_O_3_ is improved compared to the Ni foam after the oxidation and polymer coating. Ni foam’s 3D structure helps against the volume expansion. It is thought to be the primary factor behind the enhanced performance of NiO/Ni foam-based electrodes [41].

**Figure 7:**
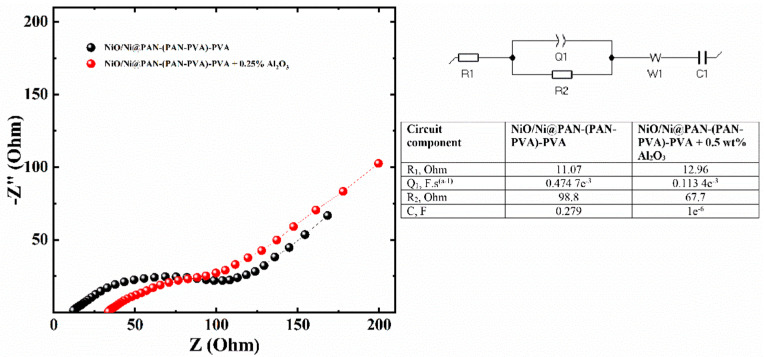
Nyquist plots of cycled NiO/Ni@PAN-(PAN-PVA)-PVA, and NiO/Ni@PAN-(PAN-PVA)-PVA with the addition of 0.5 wt% Al_2_O_3_ electrodes with the fitted circuit.

Figure 7 shows the Nyquist plots of the electrochemical impedance spectra of the NiO/Ni@PAN-(PAN-PVA)-PVA electrode and NiO/Ni@PAN-(PAN-PVA)-PVA electrode with 0.5 wt% Al_2_O_3_ after 100 cycling. Both Nyquist plots share the same characteristics, including a semicircle in the middle frequency range that is often related with charge transfer and an inclined line in the low frequency range that is responsible for lithium ion diffusion in the majority of the electrodes. According to the results of the fitted equivalent circuit, the charge transfer resistances of NiO/Ni@PAN-(PAN-PVA)-PVA and NiO/Ni@PAN-(PAN-PVA)-PVA with the addition of 0.5 wt% Al_2_O_3_ electrodes were measured to be 98.8 and 67.7, respectively. Consequently, the addition of Al_2_O_3_ nanoparticles in the GPE led to a decreased charge transfer resistance and activation energy compared to the GPE without Al_2_O_3_.

**Figure 8:**
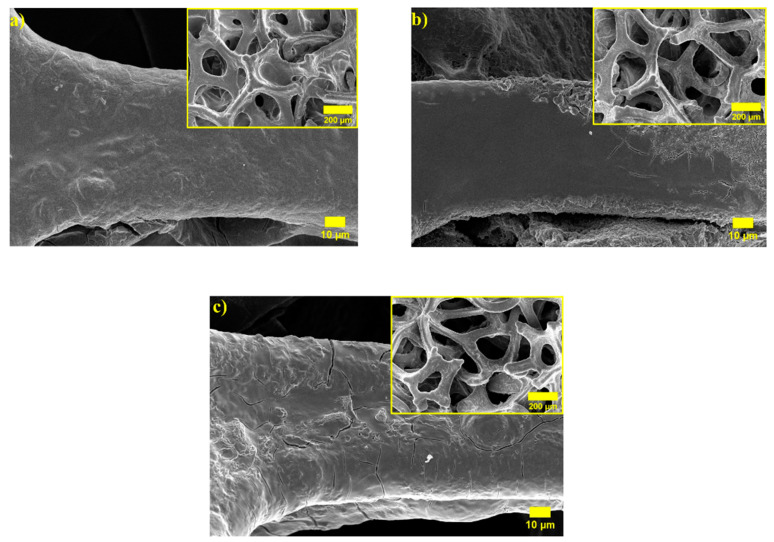
Post-mortem SEM images after 100 cycles of (**a**) NiO/Ni@PAN-(PAN-PVA)-PVA, (**b**) NiO/Ni@PAN-(PAN-PVA)-PVA with 0.25 wt% Al_2_O_3_ and (**c**) NiO/Ni@PAN-(PAN-PVA)-PVA with 0.5 wt% Al_2_O_3_.

In order to confirm the mechanical stability of Ni foam electrodes with various coating conditions after cycling coin-cells with NiO/Ni@PAN-(PAN-PVA)-PVA, NiO/Ni@PAN-(PAN-PVA)-PVA with the additions of 0.25, 0.5 wt% Al_2_O_3_ electrodes were disassembled and morphologies were investigated. Figure 8 shows the resulting images of the electrodes being retracted from the cells after 100 cycles. NiO/Ni@PAN-(PAN-PVA)-PVA electrodes retain a porous skeleton after cycling, and the polymer coating can be observed without any damage or cracks.

## 4. Conclusions

In summary, NiO/Ni@PAN-(PAN-PVA)-PVA with the addition of Al_2_O_3_ by a facile dip-coating method was successfully synthesized. Changes in surface morphology of Ni foam after thermal treatment followed by coating were revealed by SEM. A homogeneous distribution of C and Al elements throughout the coating structure was also confirmed by the EDS-SEM study at the same time.

NiO/Ni had a perfect conformal coating with PAN-(PAN-PVA)-PVA layers and even retained a porous skeleton after 100 cycles without any damage or cracks. With a high specific capacity, high coulombic efficiency, and improved structural stability, the cell demonstrated stable cycling and rate capability.

A remarkable specific discharge capacity of 546 mAh g^−1^ after 10 cycles and gradual degrease to 383 mAh g^−1^ with constant coulombic efficiency over 100 cycles were as a consequence demonstrated by the coin-cells with the GPE and the addition of 0.5 wt% Al_2_O_3_. In addition, based on the obtained results, it can be concluded that the 3D electrode’s high surface area, uniform Li^+^ diffusion, provided by the polymer coating, and unique 3D structure all contribute significantly to its improved performance.

## Data Availability

All data have been presented in this paper.
